# Decolonizing global AI governance: assessment of the state of decolonized AI governance in Sub-Saharan Africa

**DOI:** 10.1098/rsos.231994

**Published:** 2024-08-07

**Authors:** Gelan Ayana, Kokeb Dese, Hundessa Daba Nemomssa, Bontu Habtamu, Bruce Mellado, Kingsley Badu, Edmund Yamba, Sylvain Landry Faye, Moise Ondua, Dickson Nsagha, Denis Nkweteyim, Jude Dzevela Kong

**Affiliations:** ^1^ School of Biomedical Engineering, Jimma University, Jimma, Ethiopia; ^2^ Artificial Intelligence & Mathematical Modeling Lab (AIMM Lab), Dalla Lana School of Public Health, University of Toronto, 155 College St Room 500, Toronto, ON M5T 3M7; ^3^ The University of the Witwatersrand, Private Bag 3, Johannesburg, Wits 2050, South Africa; ^4^ Kwame Nkrumah University of Science and Technology (KNUST), Kumasi, Ghana; ^5^ Cheikh Anta Diop University, Avenue Cheikh Anta DIOP, Dakar SENEGAL; ^6^ The University Ngaoundere, PO Box 454, Ngaoundere. City, Adamawa Province, Cameroon; ^7^ The University of Buea, PO Box 63, Buea, South West Province, Cameroon; ^8^ Global South Artificial Intelligence for Pandemic and Epidemic Preparedness and Response Network (AI4PEP); ^9^ Africa-Canada Artificial Intelligence & Data Innovation Consortium (ACADIC)

**Keywords:** artificial intelligence, governance, decolonization

## Abstract

Global artificial intelligence (AI) governance must prioritize equity, embrace a decolonial mindset, and provide the Global South countries the authority to spearhead solution creation. Decolonization is crucial for dismantling Western-centric cognitive frameworks and mitigating biases. Integrating a decolonial approach to AI governance involves recognizing persistent colonial repercussions, leading to biases in AI solutions and disparities in AI access based on gender, race, geography, income and societal factors. This paradigm shift necessitates deliberate efforts to deconstruct imperial structures governing knowledge production, perpetuating global unequal resource access and biases. This research evaluates Sub-Saharan African progress in AI governance decolonization, focusing on indicators like AI governance institutions, national strategies, sovereignty prioritization, data protection regulations, and adherence to local data usage requirements. Results show limited progress, with only Rwanda notably responsive to decolonization among the ten countries evaluated; 80% are ‘decolonization-aware’, and one is ‘decolonization-blind’. The paper provides a detailed analysis of each nation, offering recommendations for fostering decolonization, including stakeholder involvement, addressing inequalities, promoting ethical AI, supporting local innovation, building regional partnerships, capacity building, public awareness, and inclusive governance. This paper contributes to elucidating the challenges and opportunities associated with decolonization in SSA countries, thereby enriching the ongoing discourse on global AI governance.

## 1. Introduction

The dynamic landscape of artificial intelligence (AI) is undergoing rapid transformation, heralding a new epoch characterized by unprecedented technological progress and corresponding challenges. As AI systems progressively permeate diverse facets of our existence, spanning domains such as healthcare, finance, transportation and education, it is imperative to address the pivotal matter of global AI governance. This imperative extends beyond the confines of regulatory frameworks, encompassing ethical considerations and the assurance of equitable access. The clarion call for an equitable approach to global AI governance has arguably never resonated more profoundly. To be effective and inclusive, global AI governance must embrace a decolonial approach that accords primacy to equity and facilitates opportunities for nations in the Global South to spearhead innovative solutions. Decolonial methodologies emerge as quintessential instruments in disassembling Western-centric paradigms of thought and ushering in a view of the world devoid of imperial biases [1,2].

**Figure 1.**
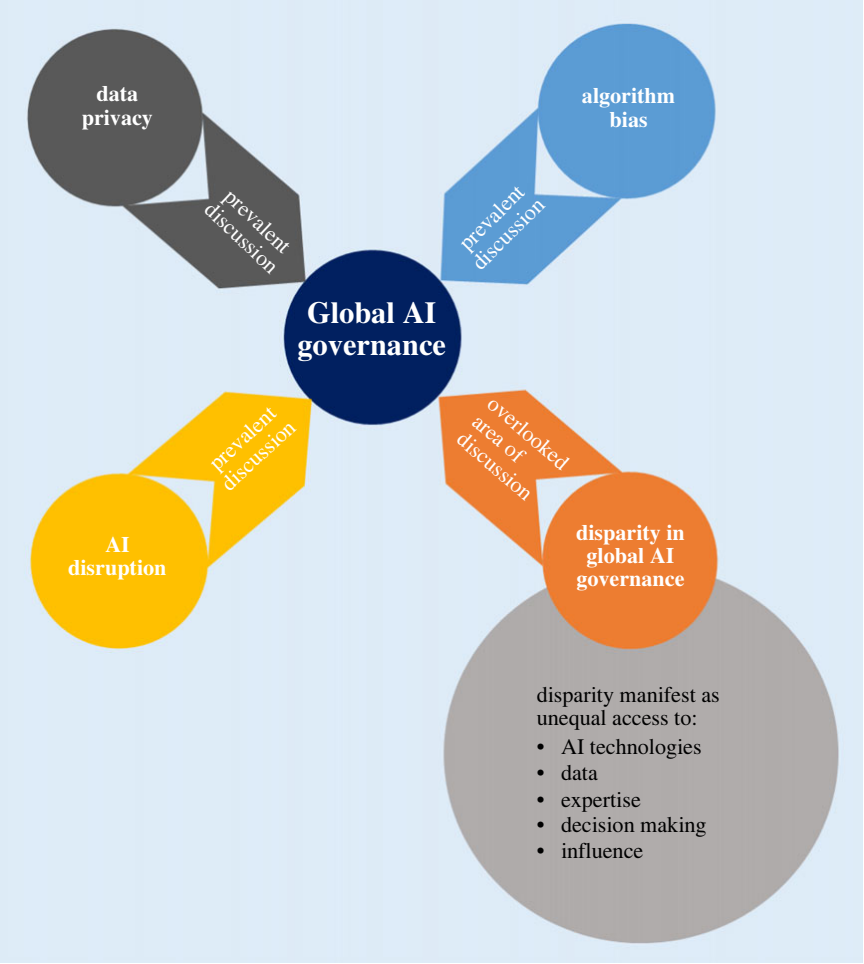
Prevalent areas of discussion in global AI governance and the overlooked issue.

In the realm of AI governance on a global scale, it is evident that prevalent discussions predominantly orbit around issues of data privacy, algorithmic bias, and the potential for AI to perturb various industries. Notwithstanding the significance of these concerns, they tend to cast a shadow over an equally pivotal issue—the disparities in AI adoption and influence. The sphere of AI governance must confront this disjunction and rectify it, thereby ensuring the just distribution of AI's benefits and repercussions across the globe. A decolonial approach to AI governance mandates an acknowledgment of the enduring legacy of colonialism, which continues to moulding our contemporary world. The enduring disparities in access to resources, education, healthcare and economic opportunities serve as palpable reminders of this historical bequest [3,4]. Within the domain of AI, these disparities manifest as unequal access to AI technologies, data, and expertise as depicted in figure 1.

The assimilation of a decolonial approach necessitates a paradigm shift in the conception and implementation of AI governance. Traditional approaches often mirror Western-centric perspectives, affording primacy to the interests of developed nations while sidelining the concerns and requisites of the Global South. A decolonial framework challenges this status quo by situating the Global South at the core of the discourse and decision-making processes, and acknowledging the idiosyncratic experiences and challenges specific to these regions. This approach underscores the value of indigenous knowledge systems and localized expertise, recognizing that AI solutions birthed in the Global North may not seamlessly apply to or account for the subtleties of issues in the Global South [5].

The elimination of biases within AI systems stands as a pivotal facet of decolonial AI governance. AI technologies often unwittingly perpetuate pre-existing inequities and biases, whether they are rooted in race, gender or geography. These biases can, in turn, circumscribe the efficacy and fairness of AI systems. A decolonial approach places emphasis on the elimination of these biases to ensure that AI technologies do not inadvertently uphold or exacerbate extant inequalities.

To fully embrace a decolonial approach, it is crucial to incorporate perspectives and voices from the Global South. This entails creating arenas for dialogue and collaboration that facilitate the co-creation of AI solutions. It is incumbent upon the custodians of AI governance to recognize the valuable insights, experiences and knowledge that the Global South contributes, thus informing the development and deployment of AI technologies. Furthermore, this approach mandates the establishment of partnerships and initiatives that ease technology transfer and bolster capacity-building in the Global South. By empowering nations in these regions to assume an active role in the development and governance of AI, the global AI ecosystem inches closer to equity [4,6,7].

Another critical dimension of decolonial AI governance is the reconfiguration of AI education. Typically, the preeminent AI education and research institutions are concentrated in the Global North, leading to the concentration of expertise within a select number of privileged regions. A decolonial approach calls for a reevaluation of this concentration, emphasizing the necessity of decentralizing AI education. This approach encourages the establishment of AI education and research centres in the Global South, ensuring that individuals from these regions have access to quality AI education and are equipped to contribute to the field. Additionally, it involves the revision of curricula to encompass diverse perspectives and to address the distinctive challenges and opportunities inherent to the Global South [8].

At its core, decolonial AI governance necessitates the deconstruction of colonial and imperial structures that perpetuate global inequalities. This entails a recognition that prevailing power dynamics within the AI domain often disenfranchise the Global South. International organizations, policy frameworks, and funding mechanisms must undergo scrutiny to ensure that they do not inadvertently perpetuate these disparities. Furthermore, a decolonial approach calls for the equitable distribution of resources and opportunities, including technology transfer, funding for AI research and development and access to AI data. It demands a reexamination of intellectual property rights to safeguard against the unjust appropriation or exploitation of knowledge and innovations originating in the Global South [6].

The ultimate aspiration of decolonial AI governance is to realign the locus of control and influence within the AI domain in favour of the Global South. This shift is not merely a matter of moral rectitude; it is a pragmatic imperative. AI technologies that are developed and governed with a global perspective inclusive of the Global South are inherently better poised to address a broader spectrum of challenges and opportunities. Nations in the Global South, marked by rich cultural diversity and distinctive challenges, can offer invaluable insights for AI solutions with broader applicability. By directly involving these nations in AI governance, the world can foster innovation, adaptability, and relevance in the development of AI technologies, thus propelling toward a more equitable and comprehensive AI landscape that serves the entirety of humanity [9].

In line with the various activities and initiatives to promote the decolonization of AI governance work of the Global South, this paper aims to assess Sub-Saharan African (SSA) countries to review and document activities supporting a decolonized way of empowerment and agency. This assessment seeks to guide stakeholders in AI on how better to support and identify opportunities for improving AI governance in a decolonized approach within the SSA countries [6,10,11]. The assessment documents AI governance structure [6,12], work, operations, and strategies to determine areas of improvement and to identify and promote the work of those demonstrating AI governance decolonization. The ultimate objective of this paper is to support, foster, and enable countries and organizations that are locally led to challenge the cause of global AI governance inequality and to promote AI governance decolonization at all levels.

### 1.1. Decolonization

Decolonization is a multifaceted and complex process that involves the dismantling of colonial structures, ideologies, and systems of oppression imposed by colonial powers on indigenous peoples and nations. It encompasses various social, political, economic, and cultural aspects aimed at restoring autonomy, sovereignty and self-determination to formerly colonized territories and populations. Understanding decolonization requires delving into its historical evolution, theories, and ongoing implications in global discourse. The concept of decolonization emerged predominantly in the twentieth century as colonies across Africa, Asia, the Caribbean and the Pacific sought independence from European imperial powers. Following World War II, the wave of decolonization intensified, fueled by anti-colonial movements, nationalist fervor, and international pressure for self-determination [13]. Iconic moments such as India's independence in 1947 and the end of European colonial rule in Africa during the 1950s and 1960s marked significant milestones in this global process [14,15]. However, decolonization did not merely entail political independence; it also entailed addressing the legacies of colonialism, including social inequalities, economic dependencies and cultural impositions, which continued to linger post-independence [16–18].

Several theories and frameworks have been developed to understand and analyze decolonization. One prominent theory is the Fanonian approach, inspired by the works of Frantz Fanon, a psychiatrist, philosopher and revolutionary from Martinique [19]. Fanon's writings, particularly in ‘The Wretched of the Earth’, emphasized the psychological, cultural, and existential dimensions of decolonization [20]. He argued that true liberation required not only political sovereignty but also the decolonization of the mind and the construction of new, independent identities. Another influential perspective is postcolonial theory, which emerged in the late twentieth century from scholars like Edward Said, Homi K. Bhabha, and Gayatri Spivak [21]. Postcolonial theory interrogates the power dynamics, cultural hegemony and epistemological implications of colonialism and seeks to deconstruct Eurocentric narratives and perspectives [22]. It highlights the ongoing effects of colonialism in shaping contemporary global relations and calls for a critical reevaluation of history, knowledge production and social justice [23]. Moreover, indigenous theories of decolonization, rooted in the experiences and struggles of indigenous peoples worldwide, emphasize the restoration of indigenous sovereignty, land rights, and self-governance [24]. These theories challenge settler colonialism, environmental degradation, and cultural erasure, advocating for indigenous resurgence and revitalization of traditional knowledge systems [25].

This paper uses the term decolonization as a concept that represents a multifaceted process of reclaiming autonomy, challenging power structures, and fostering self-determination in formerly colonized contexts. The decolonization concept's historical evolution and diverse theoretical perspectives underscore its complexity and ongoing relevance in contemporary global discourse and struggles for justice and equality [26].

### 1.2. Decolonization of science and technology systems

The decolonization of science and technology systems is a critical endeavor aimed at addressing the historical legacies of colonialism, power imbalances, and epistemic injustices embedded within scientific and technological knowledge production, dissemination, and application [27]. This transformative process seeks to challenge dominant Western-centric paradigms, center diverse knowledge systems, and promote equity, justice, and sustainability in scientific and technological endeavors [28]. Decolonization of science and technology refers to efforts to dismantle colonial hierarchies, biases and exclusions within these domains, recognizing and valuing diverse knowledge systems, perspectives and methodologies [29]. It involves reexamining and reconfiguring scientific and technological practices to be more inclusive, culturally sensitive, and responsive to the needs and aspirations of marginalized communities, particularly those disproportionately affected by colonialism and imperialism [30].

The roots of the colonization of science and technology can be traced back to colonial encounters, where Western powers imposed their scientific and technological frameworks on colonized peoples, often marginalizing indigenous knowledge systems and practices [31]. Throughout history, colonialism has been deeply intertwined with scientific exploration, exploitation of natural resources, and technological advancement, leading to the subjugation and erasure of indigenous knowledge and innovation [32]. In the 20th and 21st centuries, movements for decolonization gained momentum alongside broader struggles for independence, social justice and human rights [33]. Scholars and activists began to critique the inherent biases, Eurocentrism, and epistemic violence embedded within dominant scientific and technological paradigms [34]. These critiques highlighted the need to decolonize research methodologies, curriculum development, intellectual property rights, and institutional structures to foster greater diversity, equity and inclusion in science and technology [35].

Several theoretical frameworks inform discussions around the decolonization of science and technology [36]. One influential perspective is decolonial theory, which draws on postcolonial critiques to challenge Western-centric epistemologies and ontologies [37]. Decolonial scholars such as Walter Mignolo and Anibal Quijano argue for epistemic pluralism, recognizing multiple ways of knowing and being that have been marginalized by colonialism [38–40]. Similarly, indigenous and feminist approaches to decolonization emphasize the importance of centring indigenous and women's voices, perspectives, and knowledge systems within scientific and technological discourses [41,42]. These perspectives highlight the interconnectedness of social, cultural and ecological concerns and advocate for participatory, community-led approaches to research and innovation. Furthermore, critical race theory offers insights into the ways in which race, ethnicity, and colonialism intersect with scientific and technological practices, perpetuating systems of inequality and injustice [43]. Scholars like Glenn Adams and Phia S. Salter [44] have explored how race and power dynamics shape knowledge production, access and representation in science and technology in a perspective presented in the book ‘Seeing Race Again: Countering Colorblindness across the Disciplines' by Kimberlé Crenshaw and colleagues [43,45].

### 1.3. Historical evolution of decolonization of technology governance in the four industrial revolutions

The historical evolution of technology governance in the era of the four industrial revolutions intersects with the concept of decolonization in complex ways, reflecting broader shifts in power dynamics, knowledge production, and global socio-economic relations [46–48].

During the First Industrial Revolution (late eighteenth to early ninteenth century), the advent of mechanization, steam power, and mass production transformed manufacturing processes and spurred urbanization. Colonial powers used technological advancements to enhance their economic and military dominance over colonized territories, often exploiting indigenous resources and labour for their industrial pursuits [49]. However, the concept of decolonization was not yet prominent, as colonialism was still entrenched, and indigenous knowledge and innovation were often marginalized or appropriated by imperial forces [16,49].

The Second Industrial Revolution (late ninteenth to early twentieth century) was characterized by significant advancements in electricity, telecommunications and transportation, leading to the rise of modern infrastructure and globalization [50]. Colonial powers expanded their control over territories rich in natural resources needed for industrial production, further reinforcing colonial hierarchies and economic dependencies [48]. Nonetheless, resistance to colonial rule and calls for decolonization began to emerge, fuelled by nationalist movements, anti-colonial struggles and demands for self-determination [51].

The Third Industrial Revolution (late twentieth century to early twenty-first century), often referred to as the Digital Revolution, witnessed the proliferation of computers, telecommunications, and the internet, revolutionizing communication, commerce and information sharing [52]. Technological advancements facilitated new forms of transnational cooperation and global exchange, challenging traditional colonial power structures and fostering greater connectivity among marginalized communities. It was during this period that the concept of decolonization gained traction, with scholars and activists advocating for the recognition and empowerment of indigenous knowledge systems, cultural identities and self-governance [52,53].

In [54] the present Fourth Industrial Revolution, characterized by breakthroughs in artificial intelligence, robotics, biotechnology, and the Internet of Things, there are both opportunities and challenges for decolonization efforts [55,56]. Digital technologies enable grassroots activism, knowledge-sharing, and cultural preservation initiatives among marginalized communities, yet concerns persist about the perpetuation of digital colonialism [54,57,58]. This phenomenon sees global tech giants wielding disproportionate power over data, algorithms and digital infrastructures, exacerbating existing inequalities and marginalizing indigenous voices and perspectives [5,59]. Decolonization efforts in this era emphasize digital sovereignty, data governance and inclusive technological innovation that respects cultural diversity, indigenous rights and ethical considerations [5].

### 1.4. Decolonization of artificial intelligence governance

The decolonization of AI governance refers to the critical examination, restructuring and transformation of the policies, practices, and structures governing AI development, deployment, and regulation, with the aim of challenging colonial legacies, addressing systemic biases and centring diverse voices, perspectives, and values in decision-making processes [5,60]. It involves recognizing and redressing the historical and ongoing impacts of colonialism, imperialism and cultural hegemony on AI technologies and their governance frameworks, while promoting principles of equity, justice, accountability, and respect for human rights in AI research, development and deployment [54,59].

The evolution of the decolonization of AI governance reflects a growing recognition of the need to address biases, power imbalances, and ethical concerns embedded within AI systems and their governance structures [61]. Over time, as AI technologies have become increasingly pervasive in society, there has been a shift toward more critical engagement with how these technologies are developed, deployed and regulated [62]. Initially, discussions surrounding AI governance primarily focused on technical standards, regulatory frameworks and industry best practices, often overlooking the social and ethical implications of AI systems [63]. However, as instances of algorithmic bias, discriminatory outcomes and unequal access to AI-driven services became more apparent, there was a growing realization that traditional approaches to AI governance were inadequate [4]. The emergence of critical scholarship and social justice movements played a significant role in shaping the evolution of decolonization in AI governance [64]. Works such as ‘Data Feminism’ by Catherine D'Ignazio and Lauren F. Klein [65], ‘Algorithms of Oppression’ by Safiya Noble [65], and ‘Race After Technology’ by Ruha Benjamin [66] brought attention to the ways in which AI technologies perpetuate and exacerbate existing inequalities, particularly along lines of gender, race and socioeconomic status [67]. These critiques prompted a reevaluation of AI governance frameworks, with an increasing emphasis on principles of equity, diversity and inclusion [68]. Scholars and activists began advocating for the recognition and empowerment of marginalized communities in AI decision-making processes, as well as the incorporation of diverse perspectives and values into AI design and deployment [4]. Furthermore, indigenous approaches to AI governance have gained prominence, highlighting the importance of incorporating indigenous ways of knowing, relational ontologies and ethical principles into AI research and development [67]. Scholars such as Jason Edward Lewis and Suzanne Kite explore the potential for indigenous-led initiatives to decolonize AI governance, emphasizing the importance of respectful engagement with indigenous communities and knowledge systems [69,70].

As the field of AI governance continues to evolve, there is a growing consensus that decolonization is essential for ensuring that AI technologies serve the interests and well-being of all individuals and communities. Efforts to decolonize AI governance focus on dismantling colonial legacies, challenging dominant power structures, and centring principles of justice, equity, accountability and human dignity in AI research, development and deployment.

### 1.5. Decolonizing artificial intelligence governance and Sub-Saharan Africa

Decolonizing AI governance in SSA necessitates confronting systemic biases, power imbalances and ethical concerns inherent in AI systems and their governance structures. This endeavor mandates the active inclusion of diverse voices, perspectives and values in decision-making processes to ensure that AI technologies serve the interests and well-being of all communities. However, significant challenges persist, including data bias, limited technical capacity, and the dominance of global tech companies, which perpetuate inequalities and stifle local innovation [71]. The disparity in access to AI technologies between SSA countries and the developed Global North exacerbates these challenges [72]. For instance, services like ChatGPT offered by OpenAI are often inaccessible to the majority of SSA countries [73]. Moreover, AI tools developed for various sectors rarely incorporate data from SSA, even though they are used in the region's markets [74]. For example, breast cancer diagnosis AI tools from companies like PathAI and GE lack data collected from SSA countries, despite being available in the region [74]. Additionally, global AI governance initiatives typically exclude experts from SSA countries, neglecting the valuable insights and perspectives of those working in local contexts [75]. This is evident in bodies such as the UN AI Advisory Body (https://www.un.org/en/ai-advisory-body/members), where members predominantly hail from the Global North, failing to adequately represent the diverse voices of SSA. The lack of decision-making and influencing organizations further compounds these challenges, underscoring the urgent need for AI governance decolonization in the region. SSA countries must assess their status and explore avenues to contribute to these efforts before the situation escalates beyond manageable proportions.

## 2. Material and methods

The assessment covered 10 SSA countries: 5 of them are part of the Global South Artificial Intelligence for Pandemic and Epidemic Preparedness and Response Network (AI4PEP) (Cameroon, Ethiopia, Ghana, Senegal and South Africa) and the other 5 are the top five SSA countries according to the Government AI Readiness Index 2022 [76], excluding countries in the AI4PEP network (Kenya, Mauritius, Nigeria, Rwanda, and Seychelles). It was conducted by using information from various online resources, including government websites, reviews of available annual reports, analysis of strategy documents, social media pages and external body sources, such as funders, coalitions, networks and articles [6,9,12,77].

An assessment tool was developed and reviewed by members of the AI4PEP SSA hubs [7]. This considered the AI4PEP strategy and opportunities for decolonizing AI governance in SSA along with the three key frameworks identified by the AI4PEP network. Our adopted framework needs in the Global South contains three shells. The inner shell (how) contains the set of ethical and legal rules and codes that should be designed in such a way that they are responsible (incorporating policy and regulations), locally relevant for communities, and explainable to society at large. Moreover, they should be applied and embedded in all the processes of AI solutions in the Global South. The medium shell (what) describes the processes that should be implemented in an iterative fashion (step 1: data collection; step 2: design and development; step 3: deployment; step 4: performance; and step 5: monitoring). The third shell of our adopted framework encompasses the broader societal implications of AI deployment in the Global South. This outer shell (why) necessitates an examination of the overarching policy and regulatory frameworks guiding AI initiatives in the region, emphasizing equity, inclusivity, and community engagement. It focuses on policy and regulatory frameworks that guide AI initiatives, ensuring they are responsive to local needs and concerns while promoting transparency and accountability. This shell underscores the importance of participatory decision-making processes involving diverse stakeholders, including indigenous communities and marginalized groups, to address social dynamics and power structures. By centring their voices, AI governance frameworks can strive for solutions that are technically proficient, socially just, and inclusive.

The assessment acknowledges that AI governance that leads to meaningful results is new in SSA given the history of colonialism and being forced by circumstances to rely on the formal colonial masters. In line with the decolonizing AI governance, it is necessary to acknowledge the structural and perceived obstacles and barriers to leading AI governance for SSAs, such as limited support networks, infrastructures, limited mentorship and learning opportunities, and difficulties in having access to adequate funding and collecting data. Incorporating the guidance from AI4PEP executives, both structural (institutional structure and operations) and substantive (programmatic activities) qualities of countries were considered. Using all these, we developed five indicators which together provide evaluation of the state of decolonization of AI governance in SSA.

(I) Indicator 1: Does the country have an institution to guide AI governance?
(II) Indicator 2: Does the country have a national AI governance strategy?
(III) Indicator 3: Is ‘sovereignty’ a priority in the country's AI governance strategy?
(IV) Indicator 4: Does the country have data protection regulations?
(V) Indicator 5: Does the country necessitate or oblige the use of local data for AI tools used in the country?

Assessment criteria were defined for each of the indicators as ‘Non-existent’ with a value of 0, ‘Basic’ with a value of 0.25, ‘Moderate’ with a value of 0.5, ‘Good’ with a value of 0.75 and ‘Strong’ with a value of 1. For instance, for Indicator 1, the country was rated as ‘Non-existent’ if there is no institution for guiding AI governance. The country was rated as ‘Basic’ if the country has a dedicated body or committee or task force for AI governance, and rated as ‘Moderate’ if the country has a formally established institution for AI governance. The rating ‘Good’ was given, if the country has a formally established institution and working towards AI governance tools and guidelines, and the rating ‘Strong’ was given if the country has a formally established institution and has generated legislation or documents.

Overall evaluation was based on the average of 5 ratings of indicators. We used rounding-off in case the average is in between two overall results. Following this, the results were double-checked by triangulating information gathered from independent consultants selected from each country. The countries were then assigned a decolonized AI governance ranking as follows.

(I) Decolonization-Resistant: Accepts AI governance colonization and does not do anything.
(II) Decolonization-Blind: Ignore AI decolonization governance and do not do anything.
(III) Decolonization-Aware: Acknowledge AI governance decolonization but do not work toward decolonization.
(IV) Decolonization-Responsive: Acknowledge and consider decolonization's specific needs.
(V) Decolonization-Transformative: Address the causes of decolonization and work to transform AI governance decolonization.

According to the above overall evaluation, if a country received ‘Good’ on average, it is categorized as a ‘Decolonization-Responsive’ country.

## 3. Results

From a statistical standpoint (table 1), the findings indicate that African countries, overall, are not making significant progress in decolonizing AI governance. It is worth noting that more than half of the countries demonstrated awareness of decolonization, but if a cutoff value had been used instead of rounding off, many of these countries that are currently considered decolonization-aware might actually be classified as decolonization-blind. Out of the ten countries evaluated, only one (Rwanda) demonstrated responsiveness to decolonization efforts, while one African country was ranked as decolonization-blind (Cameroon). In general, the countries are underperforming in terms of establishing dedicated AI governance institutions and implementing the utilization of local data for their AI algorithms. However, African countries are making commendable strides in the development and enforcement of data protection regulations.

**Table 1.** Results of the state of decolonized AI governance in selected SSA countries.

country	indicator 1: institution	indicator 2: strategy	indicator 3: sovereignty as priority	indicator 4: data protection	indicator 5: use of local data	average	overall category
Cameroon [12,78]	Non-Existent (0)	Non-Existent (0)	Moderate (0.5)	Strong (1)	Basic (0.25)	Basic (0.35)	Decolonization-Blind
Ethiopia [79,80]	Moderate (0.5)	Good (0.75)	Moderate (0.5)	Moderate (0.5)	Basic (0.25)	Moderate (0.5)	Decolonization- Aware
Ghana [81,82]	Basic (0.25)	Non-Existent (0.75)	Good (0.75)	Strong (0.75)	Basic (0.25)	Moderate (0.55)	Decolonization-Aware
Senegal [83,84]	Basic (0.25)	Non-Existent (0)	Moderate (0.5)	Strong (1)	Basic (0.25)	Moderate (0.4)	Decolonization-Aware
South Africa [85–87]	Non-Existent (0)	Non-Existent (0)	Good (0.75)	Strong (1)	Good (0.75)	Moderate (0.5)	Decolonization-Aware
Kenya [88]	Basic (0.25)	Good (0.75)	Moderate (0.5)	Strong (1)	Basic (0.25)	Moderate (0.55)	Decolonization-Aware
Mauritius [89,90]	Basic (0.25)	Strong (1)	Moderate (0.5)	Strong (1)	Basic (0.25)	Moderate (0.6)	Decolonization-Aware
Nigeria [91,92]	Basic (0.25)	Moderate (0.5)	Moderate (0.5)	Strong (1)	Basic (0.25)	Moderate (0.5)	Decolonization-Aware
Rwanda [93,94]	Moderate (0.5)	Strong (1)	Good (0.75)	Strong (1)	Basic (0.25)	Good (0.7)	Decolonization-Responsive
Seychelles [12]	Basic (0.25)	Non-Existent (0)	Moderate (0.5)	Strong (1)	Basic (0.25)	Moderate (0.4)	Decolonization-Aware
Average	Basic (0.25)	Moderate (0.475)	Moderate (0.575)	Strong (0.925)	Basic (0.3)	Moderate (0.505)	Decolonization-Aware

**Figure 2.**
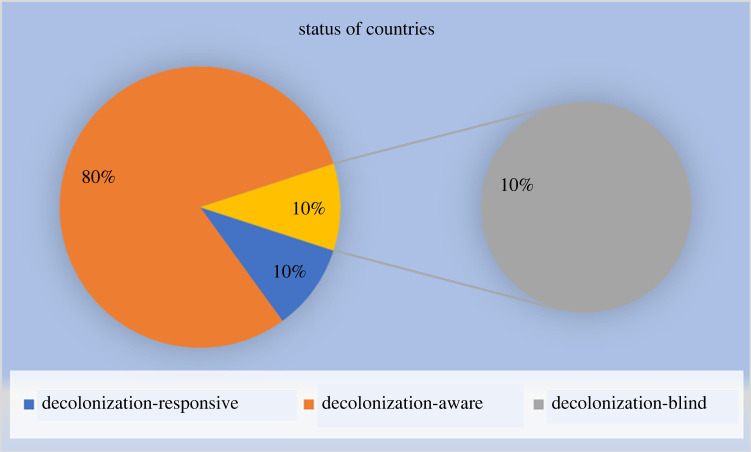
The proportion of responses regarding the state of decolonized AI governance in ten selected SSA countries.

Figure 2 below illustrates the summary of proportion of responses regarding the state of decolonized AI governance in ten selected SSA countries. As depicted in figure 2, 80% of the selected SSA countries' responses show that they are decolonization-aware, with 10% each being decolonization-responsive and decolonization-blind.

### 3.1. Evaluation by country

— Cameroon: Cameroon is undergoing both positive progress and challenges in decolonizing AI governance. The country lacks a comprehensive national AI strategy and an institution to guide AI regulation, hindering the establishment of robust frameworks for responsible AI use. Ensuring sovereignty in the AI era is also a concern, as the absence of a clear strategy and institutional framework may lead to dependence on foreign AI systems. On a positive note, Cameroon has implemented data protection regulations, but enforcement and effectiveness need improvement. Encouraging the use of local data for AI algorithms can enhance accuracy and fairness. Developing a national AI strategy, establishing an AI governance institution and promoting local data utilization are recommended to advance decolonization efforts [12,78].
— Ethiopia: Ethiopia's path towards decolonizing AI governance presents a combination of achievements and obstacles. The presence of a draft national AI strategy and an institution to guide AI regulation helps the development of robust policies. While Ethiopia has made strides in data protection regulations, enforcement requires further enhancement. The country's stance on utilizing local data for AI algorithms remains uncertain. Encouragingly, positive lessons can be drawn from their efforts in data protection. Recommendations include the speeding up of enacting a national AI strategy, strengthening of an AI governance institution and promotion of local data utilization for accurate and equitable AI systems [79,80].
— Ghana: Ghana has initiated efforts to establish institutions and frameworks dedicated to the guidance of AI governance. These forthcoming institutions are poised to assume critical roles in providing oversight, regulation, and guidance for the development and implementation of AI technologies within the country. In tandem with these institutional endeavours, the Ghanaian government is making significant investments in education and training to cultivate the requisite skills and knowledge base essential for advancing AI within its borders. To this end, the government has forged strategic partnerships with esteemed academic institutions. Ghana has acknowledged the significance of establishing a comprehensive AI governance strategy, although it is not yet fully operational. This strategic framework is designed with the overarching goal of shaping a transformed society in Ghana by the year 2033 [81]. Ghana has proactive data protection regulation which is exemplified by the enactment of the Data Protection Act in 2012. This legislative framework assumes paramount importance in the context of AI, where the preservation of personal data privacy stands as a substantive concern [81,82]. While Ghana has established data protection regulations, the rapidly evolving landscape of AI introduces unique challenges that may mandate revisions to existing laws. Such adaptations would be imperative to address issues encompassing algorithmic transparency, accountability, and equity within the context of artificial intelligence governance [82].
— Senegal: Senegal is in the process of decolonizing AI governance, with positive developments and challenges. While discussions and consultations have begun to develop a national AI strategy, formalization and implementation are still in the early stages [4]. The Minister of Communication, Telecommunications and the digital economy oversee AI technologies, but their specific focus on AI governance needs further establishment. Limited resources, infrastructure, and technical capabilities pose challenges to maintaining sovereignty in AI. Data protection regulations exist, but awareness, enforcement, and capacity building require improvement. Forcing the use of local data for AI algorithms faces obstacles due to data availability, quality, and accessibility. Recommendations include expediting the national AI strategy, building institutional capacity, enhancing data protection measures, and investing in data infrastructure. Overcoming challenges will strengthen Senegal's AI governance, assert sovereignty, and promote local data utilization [83,84].
— South Africa: South Africa (SA) has very strong regulations on data protection. However, the COVID-19 pandemic unveiled serious deficiencies in governance pertaining to data sharing. The latter includes both inter-governmental entities and the research environment. As such, SA lacks regulatory frameworks regarding data sharing and responsible AI. It is strongly recommended to engage academics and experts in the country to articulate a conversation that would eventually lead to the creation of regulatory frameworks [85–87].
— Kenya: Kenya has made significant progress in decolonizing AI governance through the implementation of national AI policies and the establishment of the Data Protection Commission. The country has emphasized the development of local AI expertise and invested in research and innovation hubs. Challenges remain in terms of resource constraints, infrastructure limitations, and the digital divide. Enhancing awareness, enforcement and capacity for data protection is crucial. Recommendations include strengthening policy implementation, investing in AI research and development, bridging the digital divide, promoting the use of local data, and fostering international collaborations [88].
— Mauritius: Mauritius is making progress in decolonizing AI governance through its national AI strategy and the establishment of the Data Protection Office. However, the country is still in the early stages of formalizing its AI strategy. Challenges include limited resources, infrastructure, and technical capabilities that affect sovereignty in AI. Data protection regulations are in place, but awareness, enforcement, and capacity building need improvement. Utilizing local data for AI algorithms is challenging due to limitations in data availability and quality. Recommendations include expediting the national AI strategy, building institutional capacity, enhancing data protection awareness and investing in data infrastructure. By addressing challenges and implementing recommendations, Mauritius can strengthen AI governance and protect data privacy [89,90].
— Nigeria: Nigeria is taking steps towards decolonizing AI governance by initiating efforts to develop a national AI strategy and engaging stakeholders. The National Information Technology Development Agency (NITDA) guides AI regulation, but its focus on AI governance is still evolving. Nigeria recognizes the importance of developing local AI expertise but faces challenges due to limited resources and infrastructure. Data protection regulations exist, but awareness, enforcement, and capacity building need improvement. Utilizing local data for AI algorithms poses challenges due to limitations in data availability and quality. Recommendations include expediting the national AI strategy, building institutional capacity, enhancing data protection awareness and investing in data infrastructure [91,92].
— Rwanda: Rwanda is actively involved in decolonizing AI governance through its comprehensive National AI Policy and Strategy. The Rwanda Utilities Regulatory Authority (RURA) guides AI regulation and has implemented guidelines for ethical standards and data protection. Rwanda emphasizes developing local AI expertise and has invested in educational programs. Data protection regulations are in place through the Personal Data Protection Law. Rwanda actively promotes the use of local data for AI algorithms through initiatives like the National Data Strategy. Challenges include effective implementation, capacity-building, and resource allocation. Recommendations include monitoring the national AI strategy, building institutional capacity, raising awareness, and fostering data sharing collaborations. Rwanda's efforts demonstrate progress in decolonizing AI governance [93,94].
— Seychelles: There is not much information on the state of decolonized AI governance in Seychelles. Seychelles should develop a comprehensive national AI strategy addressing ethics, data protection, and local data use. Establishing an institution for AI regulation is crucial to ensure compliance with ethical standards and privacy. Challenges include limited resources, infrastructure gaps, and awareness/enforcement issues. Seychelles should prioritize strategy development, capacity-building, awareness/enforcement, and collaborations [12].

**Figure 3.**
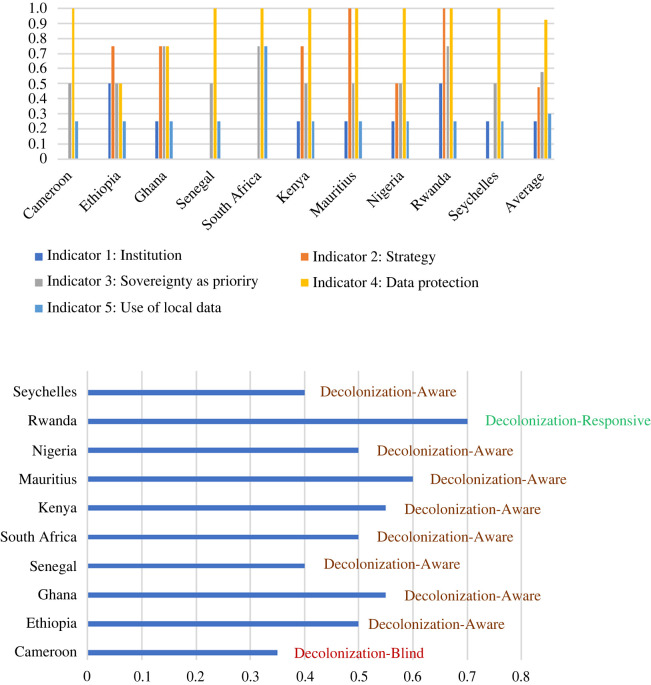
Illustration of the state of decolonized AI governance by country for SSA. (*a*) Assessment based on individual indicators. (*b*) Overall assessment of state of decolonized AI governance.

Below, in figure 3, you can find a concise overview of the status of AI governance, with a focus on decolonization, across various countries in SSA. Figure 3*a* breaks down the outcomes of individual countries concerning different decolonized AI governance indicators, while figure 3*b* presents a holistic assessment of each country's position in the context of decolonized AI governance.

## 4. Discussion

The study unveils a varied landscape of decolonizing AI governance across SSA. While most countries display awareness, substantive progress remains limited, with few demonstrating responsiveness. Challenges such as the absence of comprehensive national AI strategies, institutional frameworks and resource constraints hinder robust governance. Despite this, promising initiatives include the development of data protection regulations and investments in local AI expertise. Moving forward, expediting the implementation of national strategies, bolstering institutional capacity, promoting local data use and fostering international collaborations are crucial to advancing decolonization efforts and ensuring equitable and responsible AI governance in SSA.

Decolonizing AI governance is a complex and challenging task, but it is an important one. Here are some of the ways to decolonize AI governance:

— Involve diverse stakeholders: AI governance frameworks should be developed with the participation of a wide range of stakeholders, including people from different backgrounds, cultures, and perspectives. This will help to ensure that the frameworks are inclusive and responsive to the needs of all.
— Challenge the status quo: AI governance frameworks should challenge the status quo and seek to address the historical and systemic inequalities that have marginalized certain groups of people. This includes addressing issues such as bias, discrimination, and exclusion.
— Promote ethical AI: AI governance frameworks should promote the ethical development and use of AI. This includes ensuring that AI systems are transparent, explainable, and accountable.
— Support local innovation: AI governance frameworks should support local innovation and capacity building in Africa. This will help to ensure that Africans are at the forefront of the development and use of AI.
— Build regional partnerships: African countries can work together to develop and implement AI governance frameworks. This will help to pool resources and expertise, and to create a more unified approach to AI governance.
— Inclusion: AI governance frameworks should be inclusive and involve a wide range of stakeholders, including people from different backgrounds, cultures, and perspectives.
— Build capacity: African countries need to build capacity in the areas of data protection, privacy, transparency, explainability, necessitating local data usage, bias, discrimination, employment, security, and safety. This can be done through training programs, research, and the development of standards and guidelines.
— Promote public awareness: African countries need to promote public awareness of the issues and challenges of AI governance. This can be done through education campaigns, public consultations, and the development of educational materials.

## 5. Conclusion

In conclusion, this work advocates for a decolonial approach to global artificial intelligence governance, emphasizing equity and empowering Global South nations. It highlights the need to dismantle Western-centric cognitive frameworks and mitigate biases in artificial intelligence systems. The research evaluates Sub-Saharan African progress in adopting decolonization principles, revealing limited advancement with only Rwanda notably responsive. Most countries are deemed ‘decolonization-aware’, but concrete actions are lacking, with one country classified as ‘decolonization-blind’ among the ten countries evaluated. Recommendations include stakeholder involvement, addressing inequalities, promoting ethical artificial intelligence, supporting local innovation, building regional partnerships, capacity building, public awareness, and inclusive governance.

## Key messages

**What is already known on this topic:** Existing discussions on global artificial intelligence (AI) governance have predominantly centred on issues like data privacy and algorithmic bias, often overlooking the disparities in AI adoption. Western-centric paradigms have shaped AI governance approaches, neglecting the unique challenges faced by Sub-Saharan African (SSA) nations.

**What this study adds:** This study evaluates the decolonized AI governance progress in ten SSA countries, revealing limited advancements with Rwanda as a notable exception. It introduces a nuanced categorization of countries based on decolonization awareness and responsiveness, offering insights crucial for tailored interventions.

**How this study might affect research, practice, or policy:** The study's categorizations provide a practical framework for policymakers and practitioners to address the challenges specific to SSA countries, emphasizing stakeholder involvement, ethical AI promotion, local innovation support, and the fostering of inclusive governance for equitable AI development.

## Data Availability

All data relevant to the study are included in the article.

## References

[1] Lee D, Yoon SN. 2021 Application of Artificial Intelligence-Based Technologies in the Healthcare Industry: Opportunities and Challenges. Int. J. Environ. Res. Public Health. **18**, 271. (10.3390/ijerph18010271) 33401373 PMC7795119

[2] Davenport T, Kalakota R. 2019 The potential for artificial intelligence in healthcare. Futur. Healthc. J. **6**, 94-98. (10.7861/futurehosp.6-2-94) PMC6616181 31363513

[3] Reddy S, Allan S, Coghlan S, Cooper P. 2020 A governance model for the application of AI in health care. J. Am. Med. Inform. Assoc. **27**, 491-497. (10.1093/jamia/ocz192) 31682262 PMC7647243

[4] Belenguer L. 2022 AI bias: exploring discriminatory algorithmic decision-making models and the application of possible machine-centric solutions adapted from the pharmaceutical industry. AI ethics **2**, 771-787. (10.1007/s43681-022-00138-8) 35194591 PMC8830968

[5] Roche C, Wall PJ, Lewis D. 2022 Ethics and diversity in artificial intelligence policies, strategies and initiatives. AI Ethics **3**, 1095-1115. (10.1007/s43681-022-00218-9) PMC9540088 36246014

[6] Hassan Y. 2023 Governing algorithms from the South : a case study of AI development in Africa. AI Soc. **38**, 1429-1442. (10.1007/s00146-022-01527-7)

[7] AI4PEP. 2023 Global South AI for Pandemic & Epidemic Preparedness & Response Network. *York Univ.* See https://ai4pep.org/.

[8] Chan CKY. 2023 A comprehensive AI policy education framework for university teaching and learning. Int. J. Educ. Technol. High. Educ. **20**, 38. (10.1186/s41239-023-00408-3)

[9] Eke DO, Chintu SS. 2021 Towards shaping the future of responsible AI in Africa. Springer International Publishing.

[10] Ade-ibijola A, Okonkwo C. 2021 Artificial intelligence in Africa : emerging challenges. Springer International Publishing.

[11] Rosenstrauch D, Mangla U, Gupta A, Masau CT. 2023 Artificial Intelligence and Ethics BT - Digital Health Entrepreneurship. In (ed. A Meyers), pp. 225-239. Cham: Springer International Publishing. (10.1007/978-3-031-33902-8_16)

[12] Wakunuma K, Ogoh GI, Eke D, Akintoye S. 2022 Responsible AI, SDGs, and AI Governance in Africa. (10.23919/IST-Africa56635.2022.9845598)

[13] Chapman JM. 2023 Remaking the World: Decolonization and the Cold War. (10.5810/kentucky/9780813197487.001.0001)

[14] Dirk Moses A, Marco Duranti RB. 2020 Decolonization, self- determination, and the rise of global human rights politics. Cambridge, UK: Cambridge University Press. See https://www.dirkmoses.com/uploads/7/3/8/2/7382125/moses_-_cutting_out_the_ullcer_and_washing_away.pdf.

[15] Ibhawoh B. 2020 Seeking the political kingdom: universal human rights and the anti-colonial movement in Africa, pp. 35-53. Cambridge, UK: Cambridge University Press. (10.1017/9781108783170.003)

[16] Domínguez L, Luoma C. 2020 Decolonising Conservation Policy: How Colonial Land and Conservation Ideologies Persist and Perpetuate Indigenous Injustices at the Expense of the Environment. Land. **9**, 65. (10.3390/land9030065)

[17] Duvisac S. 2022 Decolonize! What does it mean? See https://policy-practice.oxfam.org/resources/decolonize-what-does-it-mean-621456/.

[18] Garcia-Olp M. 2018 How Colonization Impacts Identity Through the Generations: AHow Colonization Impacts Identity Through the Generations: A Closer Look at Historical Trauma and EducationCloser Look at Historical Trauma and Education. See https://digitalcommons.du.edu/cgi/viewcontent.cgi?article=2487&context=etd.

[19] Fanon F. 2019 The Problem of Blackness. *Stanford Encycl. Philos.* See https://plato.stanford.edu/entries/frantz-fanon/.

[20] Fanon F. 1963 *THE WRETCHED OF THE EARTH*. See https://www.google.com/books/edition/The_Wretched_of_the_Earth/-XGKFJq4eccC?hl=en&gbpv=1&printsec=frontcover.

[21] Go J. 2016 18Waves of Postcolonial Thought. Postcolonial Thought Soc. Theory. **59**, 108-111. (10.1093/acprof:oso/9780190625139.003.0002)

[22] Rana KS. 2021 Exploring the Elements of Postcolonialism and its Exponents. Creat. Launcher **6**, 44-52. (10.53032/tcl.2021.6.5.06)

[23] Elliott M. 2018 Indigenous Resurgence: The Drive for Renewed Engagement and Reciprocity in the Turn Away from the State. Can. J. Polit. Sci. **51**, 61-81. (10.1017/S0008423917001032)

[24] Hibbard M. 2021 Indigenous Planning: From Forced Assimilation to Self-determination. J. Plan. Lit. **37**, 17-27. (10.1177/08854122211026641)

[25] Yazzie M, Risling Baldy C. 2018 Introduction: Indigenous peoples and the politics of water. Decolonization Indig. Educ. Soc. **7**, 1-18.

[26] von Bernstorff J, Dann P. 2019 1The Battle for International Law: An Introduction. In Battle Int. Law south-north perspect. Decolonization Era. (10.1093/oso/9780198849636.003.0001)

[27] Cruz CC. 2021 Decolonizing Philosophy of Technology: Learning from Bottom-Up and Top-Down Approaches to Decolonial Technical Design. Philos. Technol. **34**, 1847-1881. (10.1007/s13347-021-00489-w)

[28] Das D. 2023 Decolonization through Technology and Decolonization of Technology. In Companion Proceedings of the 2023 ACM International Conference on Supporting Group Work, pp. 51-53. New York, NY: Association for Computing Machinery. (10.1145/3565967.3571754)

[29] Sim T. 2021 What should decolonization mean for history of science? See https://www.hps.cam.ac.uk/files/decolonise-sim.pdf.

[30] Adebisi YA. 2023 Decolonizing Epidemiological Research: A Critical Perspective. Avicenna J. Med. **13**, 68-76. (10.1055/s-0043-1769088) 37435557 PMC10332938

[31] Biermann S. 2011 Knowledge, power and decolonization: implication for non-indigenous scholars, researchers and educators. See https://researchportal.scu.edu.au/esploro/outputs/991012820482902368.

[32] Lazem S, Giglitto D, Nkwo MS, Mthoko H, Upani J, Peters A. 2022 Challenges and Paradoxes in Decolonising HCI: A Critical Discussion. Comput. Support. Coop. Work **31**, 159-196. (10.1007/s10606-021-09398-0)

[33] Sultana F. 2019 Decolonizing Development Education and the Pursuit of Social Justice. Hum. Geogr. **12**, 31-46. (10.1177/194277861901200305)

[34] Smith AWM, Jeppesen C (eds). 2017 Britain, France and the decolonization of Africa. London, UK: UCL Press. (10.2307/j.ctt1mtz521)

[35] Joseph Mbembe A. 2016 Decolonizing the university: New directions. Arts Humanit. High. Educ. **15**, 29-45. (10.1177/1474022215618513)

[36] Mohamed S, Png M-T, Isaac W. 2020 Decolonial AI: Decolonial Theory as Sociotechnical Foresight in Artificial Intelligence. Philos. Technol. **33**, 659-684. (10.1007/s13347-020-00405-8)

[37] de Sousa Santos B. 2021 Postcolonialism, Decoloniality, and Epistemologies of the South. (10.1093/acrefore/9780190201098.013.1262)

[38] Anzi A. 2021 Coloniality and its Future. Kronos **47**, 1-10. (10.17159/2309-9585/2021/v47a11)

[39] Salgado J, García-Bravo M, Benzi D. 2021 Two Decades of Aníbal Quijano's Coloniality of Power, Eurocentrism and Latin America. Context. Int. **43**, 199-222. (10.1590/s0102-8529.2019430100009)

[40] Tucker K. 2018 Unraveling Coloniality in International Relations: Knowledge, Relationality, and Strategies for Engagement. Int. Polit. Sociol. **12**, 215-232. (10.1093/ips/oly005)

[41] Manning J. 2021 Decolonial feminist theory: Embracing the gendered colonial difference in management and organisation studies. Gender, Work Organ. **28**, 1203-1219. (10.1111/gwao.12673)

[42] Datta R. 2017 Decolonizing both researcher and research and its effectiveness in Indigenous research. Res. Ethics **14**, 1-24. (10.1177/1747016117733296)

[43] Tate WF. 1997 Critical Race Theory and Education: History, Theory, and Implications. Rev. Res. Educ. **22**, 195-247. (10.2307/1167376)

[44] Adams G, Salter P. 2019 They (color) blinded Me with science: counteracting coloniality of knowledge in hegemonic psychology, pp. 271-292. Berkeley, CA: University of California Press. (10.2307/j.ctvcwp0hd.18)

[45] Crenshaw KW, Harris LC, HoSang DM, Lipsitz G. 2019 Seeing race again: countering colorblindness across the disciplines, 1st edn. Berkeley, CA: University of California Press. (10.2307/j.ctvcwp0hd)

[46] Moll I. 2022 Debunking the myth of the fourth industrial revolution (occasional paper). Johannesburg, South Africa: University of the Witwatersrand. (10.54223/uniwitwatersrand-10539-32846)

[47] Chagnon CW et al. 2022 From extractivism to global extractivism: the evolution of an organizing concept. J. Peasant Stud. **49**, 760-792. (10.1080/03066150.2022.2069015)

[48] von Tunzelmann N. 2003 Historical coevolution of governance and technology in the industrial revolutions. Struct. Chang. Econ. Dyn. **14**, 365-384. (10.1016/S0954-349X(03)00029-8)

[49] Enns C, Bersaglio B. 2020 On the Coloniality of ‘New’ Mega-Infrastructure Projects in East Africa. Antipode **52**, 101-123. (10.1111/anti.12582)

[50] Kayembe C, Nel D. 2019 Challenges and Opportunities for Education in the Fourth Industrial Revolution. African Journal of Public Affairs **11**, 79-94. (10.10520/EJC-19605d342e)

[51] Steger M. 2013 17Globalization and history: is globalization a new phenomenon? Glob. A Very Short Introd. **42**, 1056-1063. (10.1093/actrade/9780199662661.003.0002)

[52] Knell M. 2021 The digital revolution and digitalized network society. Rev. Evol. Polit. Econ. **2**, 9-25. (10.1007/s43253-021-00037-4)

[53] Pant I, Khosla S, Lama JT, Shanker V, AlKhaldi M, El-Basuoni A, Michel B, Bitar K, Nsofor IM. 2022 Decolonising global health evaluation: synthesis from a scoping review. PLOS Glob. Public Heal. **2**, e0000306. (10.1371/journal.pgph.0000306) PMC10021742 36962490

[54] Kwet M. 2019 Digital colonialism: US empire and the new imperialism in the Global South. Race Cl. **60**, 3-26. (10.1177/0306396818823172)

[55] Maynard AD. 2015 Navigating the fourth industrial revolution. Nat. Nanotechnol. **10**, 1005-1006. (10.1038/nnano.2015.286) 26632281

[56] Oosthuizen RM. 2022 The Fourth Industrial Revolution – Smart Technology, Artificial Intelligence, Robotics and Algorithms: Industrial Psychologists in Future Workplaces. Front. Artif. Intell. **5**, 913168. (10.3389/frai.2022.913168) 35875193 PMC9301265

[57] Young JC. 2019 The new knowledge politics of digital colonialism. Environ. Plan. A Econ. Sp. **51**, 1424-1441. (10.1177/0308518X19858998)

[58] Manjarrez LE. 2023 Towards pluriversal views of digital technologies: the experiences of community and indigenous radios in Chiapas, Mexico. Tapuya Lat. Am. Sci. Technol. Soc. **6**, 2254629. (10.1080/25729861.2023.2254629)

[59] Tacheva J, Ramasubramanian S. 2023 AI Empire: Unraveling the interlocking systems of oppression in generative AI's global order. Big Data Soc. **10**, 20539517231219240. (10.1177/20539517231219241)

[60] Png M-T. 2022 At the Tensions of South and North: Critical Roles of Global South Stakeholders in AI Governance. In Proceedings of the 2022 ACM Conference on Fairness, Accountability, and Transparency, pp. 1434-1445. New York, NY: Association for Computing Machinery. (10.1145/3531146.3533200) PMC10661580 37990734

[61] Adams R. 2021 Can artificial intelligence be decolonized? Interdiscip. Sci. Rev. **46**, 176-197. (10.1080/03080188.2020.1840225)

[62] Zajko M. 2021 Conservative AI and social inequality: conceptualizing alternatives to bias through social theory. AI Soc. **36**, 1047-1056. (10.1007/s00146-021-01153-9)

[63] Gianni R, Lehtinen S, Nieminen M. 2022 Governance of Responsible AI: From Ethical Guidelines to Cooperative Policies. Front. Comput. Sci. **4**, 873437. (10.3389/fcomp.2022.873437)

[64] Iazzolino G, Stremlau N. 2024 AI for social good and the corporate capture of global development. Inf. Technol. Dev. **4**, 1-18. (10.1080/02681102.2023.2299351) PMC11537297 39508029

[65] Yarrow E. 2023 Data Feminism By Catherine D'Ignazio and Lauren F. Klein, Cambridge, Massachusetts and London, England: The MIT Press, 2020. ISBN: 978-0-262-04400-4. Gender, Work Organ. **30**, 1148-1151. (10.1111/gwao.12891)

[66] Benjamin R. 2020 Review of ‘Race After Technology: Abolitionist Tools for the New Jim Code’. Soc. Forces **98**, 1-3. (10.1093/sf/soz162)

[67] Noble SU. 2018 Algorithms of oppression: How search engines reinforce racism. New York, NY: Nework University Press. (10.2307/j.ctt1pwt9w5) 34709921

[68] Wolbring G, Nguyen A. 2023 Equity/equality, diversity and inclusion, and other EDI phrases and EDI policy frameworks: a scoping review. Trends High. Educ. **2**, 168-237. (10.3390/higheredu2010011)

[69] Arista N, Costanza-Chock S, Ghazavi V, Kite S. 2021 Against reduction: designing a human future with machines. Cambridge, MA: MIT Press. (10.7551/mitpress/14157.001.0001)

[70] Biddle JL, Hibberd L. 2023 The Artificial as an Intelligent Indigenous/Indigenizing System. Vis. Anthropol. Rev. **39**, 458-474. (10.1111/var.12284)

[71] Dwivedi YK et al. 2023 Opinion Paper: ‘So what if ChatGPT wrote it?’ Multidisciplinary perspectives on opportunities, challenges and implications of generative conversational AI for research, practice and policy. Int. J. Inf. Manage. **71**, 102642. (10.1016/j.ijinfomgt.2023.102642)

[72] Arakpogun E, Elsahn Z, Olan F, Elsahn F. 2021 Artificial intelligence in Africa: challenges and opportunities, pp. 375-388. Cham, Switzerland: Springer. (10.1007/978-3-030-62796-6_22)

[73] Maslej N et al. 2023 Artificial intelligence index report 2023. *arXiv Prepr. arXiv2310.03715*. (10.48550/arXiv.2310.03715)

[74] Eke DO, Chintu SS, Wakunuma K. 2023 Towards Shaping the Future of Responsible AI in Africa BT - Responsible AI in Africa: Challenges and Opportunities. In (eds DO Eke, K Wakunuma, S Akintoye), pp. 169-193. Cham: Springer International Publishing. (10.1007/978-3-031-08215-3_8)

[75] Okolo CT, Aruleba K, Obaido G. 2023 Responsible AI in Africa—Challenges and Opportunities BT - Responsible AI in Africa: Challenges and Opportunities. In (eds DO Eke, K Wakunuma, S Akintoye), pp. 35-64. Cham: Springer International Publishing. (10.1007/978-3-031-08215-3_3)

[76] Rogerson A, Hankins E, Nettel PF, Rahim S. 2022 Government AI readiness index 2022. Oxford, UK: Oxford Insights. See https://www.unido.org/sites/default/files/files/2023-01/Government_AI_Readiness_2022_FV.pdf.

[77] Butcher N, Wilson-strydom M, Baijnath M. 2021 Artificial intelligence capacity in sub- artificial intelligence capacity in sub-saharan. See https://idl-bnc-idrc.dspacedirect.org/server/api/core/bitstreams/8e7f2bb6-1079-4052-b5b8-7a26feced2cf/content.

[78] Nwenfor BE. 2023 Cameroon to Get First Artificial Intelligence Centre worth FCFA 1.3 billion. *Pan-African Visions*. See https://panafricanvisions.com/2019/08/cameroon-to-get-first-artificial-intelligence-centre-worth-fcfa-1-3-billion/.

[79] Bilali H. 2023 Ethiopia nears completion of national AI policy. See https://www.wearetech.africa/en/fils-uk/news/ethiopia-nears-completion-of-national-ai-policy.

[80] EAII. 2020 Ethiopian Artificial Intelligence Institute. See https://www.aii.et/about-us/.

[81] Suuk M. 2023 Ghana debates regulating artificial intelligence. *DW*. See https://www.dw.com/en/maxwell-suuk/person-35992797.

[82] Legal Insights. 2023 Artificial Intelligence in Ghana. See https://integrisa.com/insights/f/artificial-intelligence-in-ghana.

[83] Smart Africa. 2023 Senegal unveils its national data strategy. See https://smartafrica.org/senegal-unveils-its-national-data-strategy/.

[84] Heng S, Tsilionis K, Scharff C, Wautelet Y. 2022 Understanding AI ecosystems in the Global South : The cases of Senegal and Cambodia. Int. J. Inf. Manage. **64**, 102454. (10.1016/j.ijinfomgt.2021.102454)

[85] DataGuidance O. 2022 South Africa - Data Protection Overview. See https://www.dataguidance.com/notes/south-africa-data-protection-overview.

[86] 2022 Ai in South Africa. *Obs. OECD Policy*. See https://oecd.ai/en/dashboards/countries/SouthAfrica.

[87] Wentzel W. 2022 Data Protected - South Africa. *Linklaters*. See https://www.linklaters.com/en/insights/data-protected/data-protected—south-africa.

[88] Kayla EK. 2023 The Art of AI in Kenya: Nurturing Innovation, Policy, and Ethical Progress. *LinkedIn*. See https://www.linkedin.com/pulse/art-ai-kenya-nurturing-innovation-policy-ethical-edward-kip-kalya/.

[89] Gilbert P. 2020 Mauritius, Egypt and SA are Africa's most AI ready. *Connect. Africa*. See https://www.connectingafrica.com/author.asp?section_id=761&doc_id=765789.

[90] Commonwealth T. 2018 Mauritius Artificial Intelligence (AI) Strategy<MM. See https://tradecca.thecommonwealth.org/document/mauritius-artificial-intelligence-ai-strategy-mm.

[91] Nwaokocha A. 2023 Nigeria invites global experts to collaborate on national AI strategy. *Cointelegraph*. See https://cointelegraph.com/news/nigeria-calls-on-global-experts-for-national-ai-strategy-co-creation.

[92] Digwatch. 2023 Nigerian government calls on researchers to collaborate on national AI strategy. See https://dig.watch/updates/nigerian-government-calls-on-researchers-to-collaborate-on-national-ai-strategy.

[93] Magoro J. 2023 Access Alert | Rwandan government approves National AI Policy. *Partinership, Access*. See https://accesspartnership.com/access-alert-rwandan-government-approves-national-ai-policy/.

[94] Society TF. 2023 The Development of Rwanda's National Artificial Intelligence Policy. See https://thefuturesociety.org/development-of-rwandas-national-artificial-intelligence-policy/.

